# Gremlin-1 Induces BMP-Independent Tumor Cell Proliferation, Migration, and Invasion

**DOI:** 10.1371/journal.pone.0035100

**Published:** 2012-04-13

**Authors:** Minsoo Kim, Soomin Yoon, Sukmook Lee, Seon Ah Ha, Hyun Kee Kim, Jin Woo Kim, Junho Chung

**Affiliations:** 1 Department of Biochemistry and Molecular Biology, Seoul National University College of Medicine, Chongno-gu, Seoul, Republic of Korea; 2 Department of Cancer Biology, Seoul National University College of Medicine, Chongno-gu, Seoul, Republic of Korea; 3 Cancer Research Institute, Seoul National University College of Medicine, Chongno-gu, Seoul, Republic of Korea; 4 Scripps Korea Antibody Institute, Chuncheon-si, Gangwon-do, Republic of Korea; 5 Molecular Genetic Laboratory, College of Medicine, The Catholic University of Korea, Seocho-ku, Seoul, Republic of Korea; 6 Department of Obstetrics and Gynecology, College of Medicine, The Catholic University of Korea, Seocho-ku, Seoul, Republic of Korea; University of Texas MD Anderson Cancer Center, United States of America

## Abstract

Gremlin-1, a bone morphogenetic protein (BMP) antagonist, is overexpressed in various cancerous tissues but its role in carcinogenesis has not been established. Here, we report that gremlin-1 binds various cancer cell lines and this interaction is inhibited by our newly developed gremlin-1 antibody, GRE1. Gremlin-1 binding to cancer cells was unaffected by the presence of BMP-2, BMP-4, and BMP-7. In addition, the binding was independent of vascular endothelial growth factor receptor-2 (VEGFR2) expression on the cell surface. Addition of gremlin-1 to A549 cells induced a fibroblast-like morphology and decreased E-cadherin expression. In a scratch wound healing assay, A549 cells incubated with gremlin-1 or transfected with gremlin-1 showed increased migration, which was inhibited in the presence of the GRE1 antibody. Gremlin-1 transfected A549 cells also exhibited increased invasiveness as well as an increased growth rate. These effects were also inhibited by the addition of the GRE1 antibody. In conclusion, this study demonstrates that gremlin-1 directly interacts with cancer cells in a BMP- and VEGFR2-independent manner and can induce cell migration, invasion, and proliferation.

## Introduction

Gremlin-1 is a 20.7-kDa protein consisting of 184 amino acids with a cysteine-rich region, a cysteine knot motif, and a structure shared by members of the TGF-β superfamily. This protein is evolutionarily conserved and the human gremlin gene (*GREM1*) has been mapped to chromosome 15q13-q15 [1], [2]. Gremlin-1 is a secreted protein and three isoforms have been reported [3]. Isoform 1 is the most common isoform and isoforms 2 and 3 have deletions of amino acids 39–79 and 10–79, respectively. Gremlin-1 forms heterodimers with BMP-2, BMP-4, and BMP-7 and thus inhibits their binding to receptors on the cell surface [4]–[6]. In addition, Gremlin-1 plays an important role in regulating BMPs during lung, limb, and kidney development as well as during neural crest cell differentiation [7], [8]. In addition to its antagonistic effect on soluble ligands, gremlin-1 interacts intracellularly with the BMP-4 precursor protein and downregulates BMP-4-mediated signaling activity in embryonic lungs [9]. Gremlin-1 also interacts with Slit proteins, a family of secreted axonal guidance proteins, and acts as an inhibitor of monocyte chemotaxis [10]. Recently it was reported that gremlin-1 binds vascular endothelial growth factor receptor-2 (VEGFR2) in a BMP-independent manner and modulates angiogenesis [11]. Gremlin-1 is overexpressed in various human tumors including carcinomas of the cervix, endometrium, lung, ovary, kidney, breast, colon, and pancreas [12], [13] but its role in carcinogenesis has not been studied in detail.

In this study, we report that gremlin-1 directly binds to the cancer cell lines A549, HeLa, A172, and A431. In A549 cells, gremlin-1 induces cell migration, proliferation, and invasion. The interaction with cancer cells was not mediated by VEGFR2, the only known cell surface receptor of gremlin-1, and was unaffected by the presence of BMPs. Gremlin-1-transfected A549 cells showed increased tumor growth *in vivo*, suggesting that gremlin-1 overexpression may play a role in tumorigenesis.

## Materials and Methods

### Cell culture

A549, HeLa, A172, and A431 cells were obtained from the Korean Cell Line Bank (Seoul, Republic of Korea) and human umbilical vein endothelial cells (HUVECs) were obtained from Invitrogen (Carlsbad, CA). A549, A172 and A431 cells were grown in RPMI-1640 media (Welgene, Seoul, Korea) supplemented with 10% FBS (GIBCO, Grand Island, NY). HeLa cells were cultured in MEM media (Welgene) supplemented with 10% FBS. HUVECs were cultured in endothelial cell growth media-2 (EGM-2, Lonza, Walkersville, MD).

### Cell transfection

A549 cells (5.0×10^5^ cells) were plated 1 day prior to transfection to achieve 70% confluency at the time of transfection. The gremlin cDNA was amplified from a human cervical tissue cDNA library as described previously [12]. *Hind*III and *Xho*I restriction sites were introduced at the 5′ and 3′ ends, respectively, using the following PCR primers: 5′ CCC AAG CTT ATG AGC CGC ACA GCC TAC AC 3′ and 5′ CCG CTC GAG ATC CAA ATC GAT GGA TAT GC 3′. The PCR product was digested with *Hind*III and *Xho*I and then ligated into the pcDNA3.1/*myc*-His vector (Invitrogen). This expression vector was transfected into cells using the Lipofectamine 2000 reagent (Invitrogen) according to the manufacturer's instructions. To transfect a 100 mm dish of A549 cells, 24 µg of plasmid was mixed with 60 µl of Lipofectamine. Antibiotic selection was performed using 1.0 mg/ml of G418 (Invitrogen). The selected cells are termed gremlin-1-A549 cells. In a parallel experiment, A549 cells were transfected with pcDNA3.1/*myc*-His vector alone and used as mock-A549 cells.

### Expression and purification of gremlin-1

Gene encoding human gremlin-1 and human IgG1-Fc fusion protein was constructed using overlapping PCR as described previously [15]. The linker primer sequences (forward and backward for gremlin-1) are as follows; 5′ GGC CCC ACC GGC CCC ATC CAA ATC GAT 3′, and 5′ GGG GCC GGT GGG GCC TCG GGT GGC GGT GGC 3′. The linker primer sequences forward and backward for human IgG1-Fc are as follows; 5′ AAG CTT GTG GCC CAG GCG GCC ATG AGC CGC ACA GCC TAC 3′, and 5′ GGA TCC TCA TTT TGG CGG GGA CAG GGA GAG 3′. The PCR products were digested with *Hind*III and *BamH*I and cloned into the pCEP4 expression vector (Invitrogen). HEK293F cells (Invitrogen) were cultured in GIBCO FreeStyle™ 293 Expression media (Invitrogen) at a cell density between 0.1×10^6^ and 2.0×10^6^ cells/ml. Cells were grown in disposable Erlenmeyer tissue culture flasks with vented caps (Corning Inc.) at 135 rpm on an orbital shaking incubator (37°C, 8% CO_2_, Minitron, INFORS HT, Switzerland). One day prior to transfection, cell cultures were diluted with fresh media to achieve a density of 1.0×10^6^ cells/ml, which resulted in a density of 2.0×10^6^ cells/ml on the day of transfection. HEK293F cells were transfected with pCEP4 expression vector using Lipofectamine 2000 (Invitrogen) according to the manufacturer's instructions. Transfected cells were again cultured in the orbital shaking incubator and culture supernatants were harvested the third day after transfection. The gremlin-1-Fc fusion protein was purified using protein A affinity gel chromatography as described previously [15].

### Generation of gremlin-1 antibody (GRE1)

New Zealand white rabbits were immunized with gremlin-1-Fc and a rabbit immune library was constructed using total RNA prepared from the bone marrow and spleen of the immunized rabbits, as described previously [14]. Single-chain variable fragment (scFv) clones were selected from the library using phage display as described previously [15]. The scFv fragments were converted to full length IgG and overexpressed as described previously [16]. The specificity of the GRE1 antibody was determined using western blot analyses (Fig. S1).

### RNA isolation and RT-PCR

Total RNA was isolated from A549 cells, HeLa cells, and HUVECs using the TRIzol reagent (Invitrogen) according to the manufacturer's instructions. cDNA was synthesized using the Superscript® III First-Strand Synthesis system (Invitrogen).

The primer sequences were as follows: VEGFR-2 forward: 5′-TGATCGGAAATGACACTGGA-3′, VEGFR-2 reverse: 5′- TGCTTCACAGAAGACCATGC-3′, gremlin-1 forward: 5′-AACAGTCGCACCATCATCAA-3′, gremlin-1 reverse: 5′-AATTTCTTGGGCTTGCAGAA-3′, GAPDH forward: 5′-AGGTGAAGGTCGGAGTCAACG-3′, GAPDH reverse: 5′-AGGGGTCATTGATGGCAACA-3′. The PCR mixtures were prepared according to the manufacturer's instructions with PCR conditions of 35 cycles of 30 sec at 94°C, 30 sec at 56°C, and 1 min at 72°C on a 2720 Thermal Cycler (Applied Biosystems, Foster City, CA).

### Flow cytometry

Adherent cells were trypsinized and washed with 1% (w/v) BSA in phosphate-buffered saline (PBS). Suspension cells were collected by centrifugation at 500×*g* for 2 min and washed with 1% (w/v) BSA in PBS. All cells were incubated with His-tagged gremlin-1 (R&D Systems, Minneapolis, MN) at a final concentration of 100 nM in 1% (w/v) BSA in PBS for 1 h at 37°C. The cells were then washed twice with 1% (w/v) BSA in PBS and incubated for 30 min at 37°C in the dark with a FITC-conjugated His antibody (Abcam, Cambridge, UK) at a final concentration of 5 µg/ml. Cells were then washed twice with 1% (w/v) BSA in PBS and resuspended in 500 µl of PBS prior to analysis on a FACSCanto II flow cytometer (BD Biosciences, San Jose, CA).

To determine the neutralizing efficacy of the gremlin-1 antibody GRE1, cells were incubated with 100 nM of His-tagged gremlin-1 and 10 µM of GRE1 in 1% (w/v) BSA in PBS for 1 h at 37°C and probed with a FITC-conjugated His antibody (Abcam).

A549 cells were treated with 1 µM of BMP-2, BMP-4, or BMP-7 (R&D Systems, Minneapolis, MN) and 100 nM of gremlin-1-Fc simultaneously and incubated for 1 h at 37°C. Cells were probed with FITC-conjugated IgG-Fc specific antibody (5 µg/ml, Invitrogen). Cells were then analyzed on a FACSCanto II flow cytometer.

### Western blot analyses

HUVECs, A549 cells, and HeLa cells were lysed in ice-cold lysis buffer [50 mM Tris-HCl (pH 7.4), 150 mM NaCl, 2 mM EDTA, 1% Triton-X 100, 0.1% SDS, 1 mM PMSF] containing a protease inhibitor cocktail (Sigma-Aldrich, St. Louis, MO). Western blots were performed as described previously [14]. E-cadherin (1∶1,000 dilution; Abcam), VEGFR-2 (1∶1,000 dilution; Cell Signaling Technology, Danvers, MA), and β-actin (1∶10,000 dilution; Applied Biological Materials, Richmond, BC) antibodies were used as the primary antibodies. The secondary antibodies were horseradish peroxidase (HRP)-conjugated anti-mouse IgG (1∶1,000 dilution; Pierce Chemical Co., Rockford, IL) or HRP-conjugated anti-rabbit IgG (1∶1,000 dilution; Pierce Chemical Co.). Blots were visualized using an enhanced chemiluminescence system (Pierce) per the manufacturer's instructions.

To analyze E-cadherin expression, A549 cells (1.0×10^5^ cells/well) were seeded onto a 60-mm dish and grown to 50% confluence. Cells were treated with 100 nM of His-tagged gremlin-1 for 3 days. Cells were lysed and analyzed by western blot as described above.

To determine the neutralizing efficacy of the gremlin-1 antibody GRE1, gremlin-1-A549 cells and mock-A549 cells (1.0×10^5^ cells/well) were seeded onto a 60-mm dish to 50% confluence. Mock-A549 cells were cultured without treatment and Gremlin-1-A549 cells were cultured for 24 h in the presence of 10 µM GRE1 or control antibody (Palivizumab, Synagis, Abbott Laboratories, Abbott Park, IL). Cells were lysed and analyzed by western blot as described above.

To analyze gremlin-1 expression, the culture supernatants from mock-A549 and gremlin-1-A549 cells were resolved by SDS-PAGE as described above. The blots were incubated for 1 h at room temperature with HRP-conjugated -His antibody (1∶1000 dilution, R&D Systems). Blots were visualized using an enhanced chemiluminescence system (Pierce) per the manufacturer's instructions.

### Enzyme immunoassay

Microtiter plates (Corning Costar Corp., Cambridge, MA) were coated with 100 nM of BMP-2, BMP-4, or BMP-7 (R&D Systems) and blocked with 1% (w/v) skim milk in PBS. Gremlin-1-Fc (10 nM) or gremlin-1-Fc (10 nM) plus 500 nM of GRE1 antibody were added to the wells. After washing, plates were incubated with an HRP-conjugated IgG-Fc specific antibody (1∶5,000 dilution; Pierce Chemical Co.). 2,2′-azino-bis(3-ethylbenzothiazoline-6-sulphonic acid (ABTS) substrate solution (Amresco, Solon, OH) was used for the coloring reaction as described previously [17]. Experiments were performed in triplicate.

### Crystal violet staining assay

A549 cells were seeded in 24-well plates (1.0×10^4^ cells/well) and treated with 100 nM of His-tagged gremlin-1 for 3 days. Media was removed and cells were washed with PBS and fixed with 4% paraformaldehyde in PBS for 10 min. Cells were stained with 0.05% crystal violet in distilled water for 30 min. The staining solution was removed and the cells were washed 3 times with PBS as described [18]. Images were obtained using a Leica DFL290 camera (Leica Microsystems, Wetzlar, Germany) and analyzed using Leica application suite software (Leica Microsystems)

### Immunofluorescence staining

A549 cells (1.5×10^4^ cells/well) were seeded on glass coverslips coated with poly-L-lysine (100 µg/ml, Sigma) and grown to 50% confluence. Cells were treated with 100 nM of His-tagged gremlin-1 for 3 days, rinsed in PBS, and fixed in 4% paraformaldehyde in PBS for 30 min at room temperature. Fixed cells were permeabilized with 0.2% Triton X-100 in PBS (PBST) at room temperature for 10 min and then blocked with 1% gelatin in PBST for 30 min at room temperature. Immunofluorescent staining was performed using an E-cadherin antibody (Abcam) followed by an Alexa 488-conjugated secondary antibody (Invitrogen). Nuclei were stained with DAPI (1∶1,000 dilution; Invitrogen) and actin filaments were stained using rhodamine-phalloidin (1∶1,000 dilution; Invitrogen). Coverslips were mounted on glass slides using aqueous mounting medium with anti-fading agents (Biomeda Corp., Foster City, CA). Images were acquired using a LSM 5 PASCAL Laser Scanning Microscope (Carl Zeiss, Germany) and analyzed using LSM 5 PASCAL software.

### Cell migration assay

Cells were seeded in 24-well plates at a density of 1.0×10^5^ cells per well. A scratch wound was generated by scratching with a pipette tip. After rinsing with media to remove detached cells, 100 nM of His-tagged gremlin-1 was added to the cultures for 24 h. Photographic images were taken from each well immediately and again after 24 h using a Leica DFL290 camera (Leica Microsystems). Images were analyzed using Leica application suite software (Leica Microsystems). The distance that cells migrated through the area created by scratching was determined by measuring the wound width at 24 h and subtracting it from the wound width at the start. The values obtained were then expressed as % migration, setting the migrating distance of cells untreated as 100% as described [19].

To determine the neutralizing efficacy of the gremlin-1 antibody GRE1, scratched cells were incubated for 24 h with His-tagged gremlin-1 alone or plus 10 µM GRE1 (or 10 µM control antibody). The distance was determined as described above.

Using the same protocol, mock transfected A549 cells and gremlin-1 transfected A549 cells were seeded and scratched. Mock-A549 cells were cultured without any treatment while Gremlin-1-A549 cells were cultured for 24 h in the presence of 10 µM GRE1 or control antibody. The distance was determined as described above. The results were representative of three independent experiments.

### Cell invasion assay

Cell invasion assays were performed using ECM coated inner chambers (Chemicon, Temecula, CA) per the manufacturer's instructions. Mock-A549 cells and gremlin-1-A549 cells (3.0×10^5^ cells per well) were suspended in 300 µl of serum-free media. Complete media (500 µl) containing 10% FBS was added to the bottom wells of the plate. Cells were incubated for 48 h. Non-migrating cells were wiped away and washed with PBS. The membranes were fixed with 4% paraformaldehyde in PBS and stained with a crystal violet stain solution (Chemicon). Images were acquired using a Leica DFL290 camera (Leica Microsystems) and analyzed using Leica application suite software. Migrated cells were counted in four separated fields per well. The values obtained were then expressed as % invasion, setting the cell counts of mock-A549 cells as 100%. The results were representative of three independent experiments.

### Cell proliferation assay

Cell proliferation was determined using CellTiter 96 Aqueous One Solution Cell Proliferation Assay (Promega, Madison, WI) following the manufacturer's protocol. Experiments were performed in 96-well plates in RPMI-1640 media supplemented with 10% FBS. Mock-A549 cells and gremlin-1-A549 cells were seeded at a density of 1,000 cells per well. After 24 h, cells were washed twice with serum-free media and cultured in 100 µl of complete media with or without 3 µM of GRE1 antibody. Cell proliferation was determined by using Labsystems Multiskan Ascent Photometric plate reader (Thermo Labsystems, Franklin, MA) for a 96 well plate with a 492 nm filter. Experiments were performed in triplicate.

### In vivo tumorigenesis

All animal experiments were authorized by the Institute of Laboratory Animal Resources Seoul National University and Use Committee (Permit number: SNU-11-0207). Gremlin-1-A549 cells and mock-A549 cells (1.0×10^6^ cells/mouse) were injected subcutaneously in the right flank of 4- to 6 week-old female, athymic nude mice (7 mice in each treatment group). Tumor formation and size were assessed weekly by caliper measurements of the length and width of the tumors. Tumor volumes were calculated using the following formula: (Length×Width×Height)/2 [20].

### Statistical analyses

Statistical significance was determined using a Student's t-test and P values<0.05 were considered statistically significant. All statistical tests were performed using SPSS version 17.0 software (SPSS, Chicago, IL).

## Results

### Gremlin-1 directly interacts with human cancer cell lines

**Figure 1:**
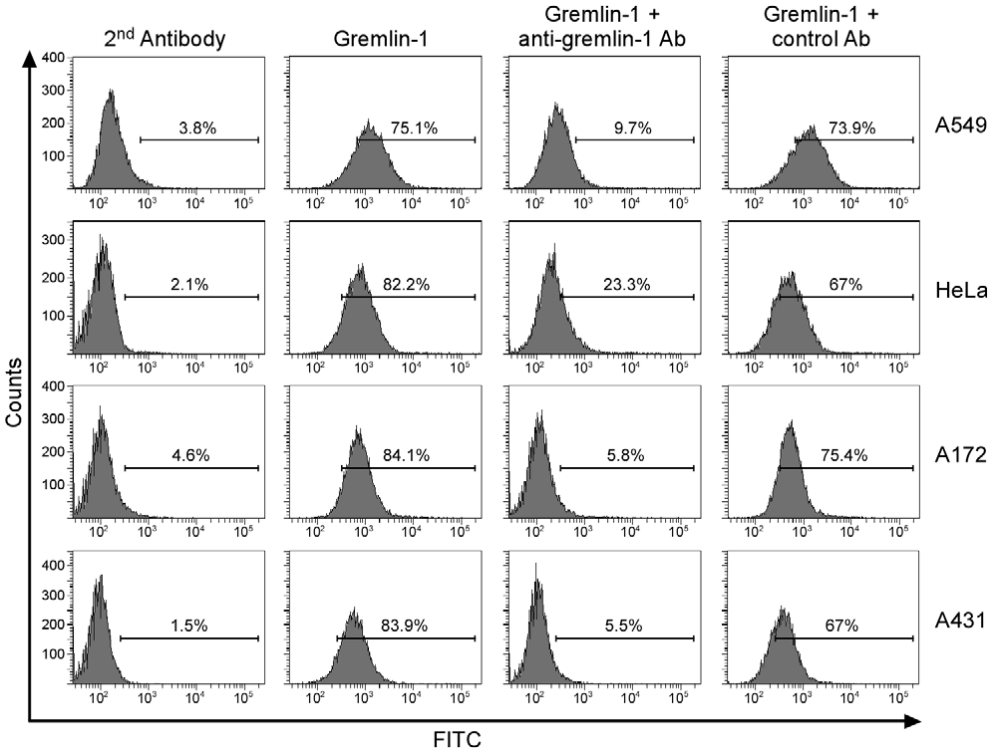
Gremlin-1 interacts with human cancer cell lines. Cells were incubated with gremlin-1, in the presence or absence of the neutralizing antibody GRE1 as described. The four cancer cell lines interacted directly with gremlin-1 and this interaction was inhibited upon the addition of the neutralizing antibody GRE1.

**Figure 2:**
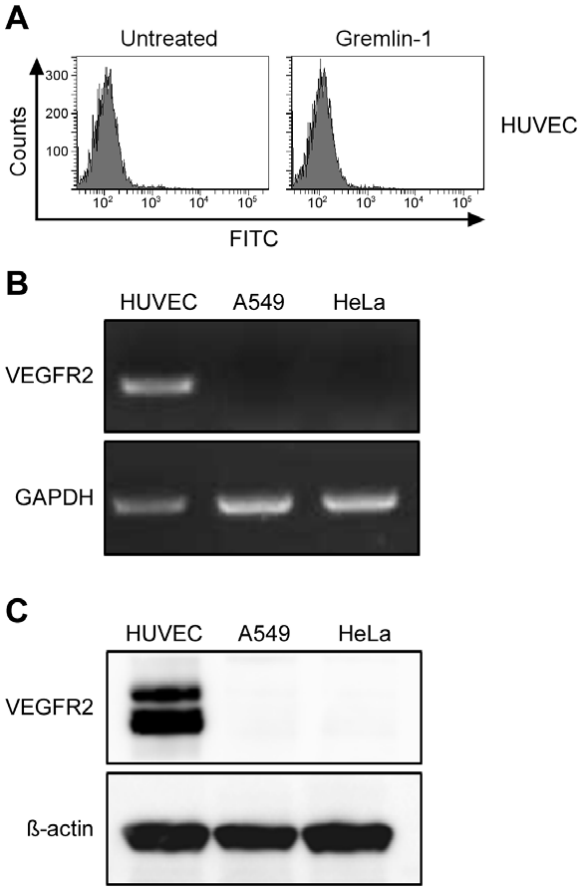
The interaction of gremlin-1 with cancer cells is independent of VEGFR2 expression. (A) Gremlin-1 does not interact with HUVECs as measured by flow cytometry. (B) RT-PCR analysis of VEGFR2 mRNA indicates the presence of VEGFR2 in HUVECs but not in A549 or HeLa cells. (C) Immunoblot analysis using a VEGFR2 antibody indicates that A549 and HeLa cells do not express VEGFR2.

The interaction of gremlin-1 with cancer cell lines was analyzed by flow cytometry. Four cancer cell lines, including A549 cells, interacted with gremlin-1 (Fig. 1). The gremlin-1 antibody GRE1 inhibited the binding of gremlin-1 to all cell lines, including A549 (Fig. 1). These data indicate that gremlin-1 directly interacts with cancer cells depending on a motif either co-localized with the epitope of antibody GRE1 or affected by the binding of GRE1. Next, we evaluated whether the interaction of gremlin-1 with the cell lines was mediated by VEGFR2, the only known cell surface receptor of gremlin-1 [11]. In HUVECs, the binding of gremlin-1 was not significant (Fig. 2A) but VEGFR2 mRNA and protein expression was confirmed using RT-PCR and immunoblot analyses (Fig. 2B and 2C). However, although gremlin-1 interacted with A549 and HeLa cells (Fig. 1), VEGFR2 mRNA and protein were not detected in these cells as measured by RT-PCR and immunoblot analyses (Fig. 2B and 2C). Therefore, we conclude that gremlin-1 can interact with cancer cells directly and this interaction does not have to be mediated by VEGFR2.

**Figure 3:**
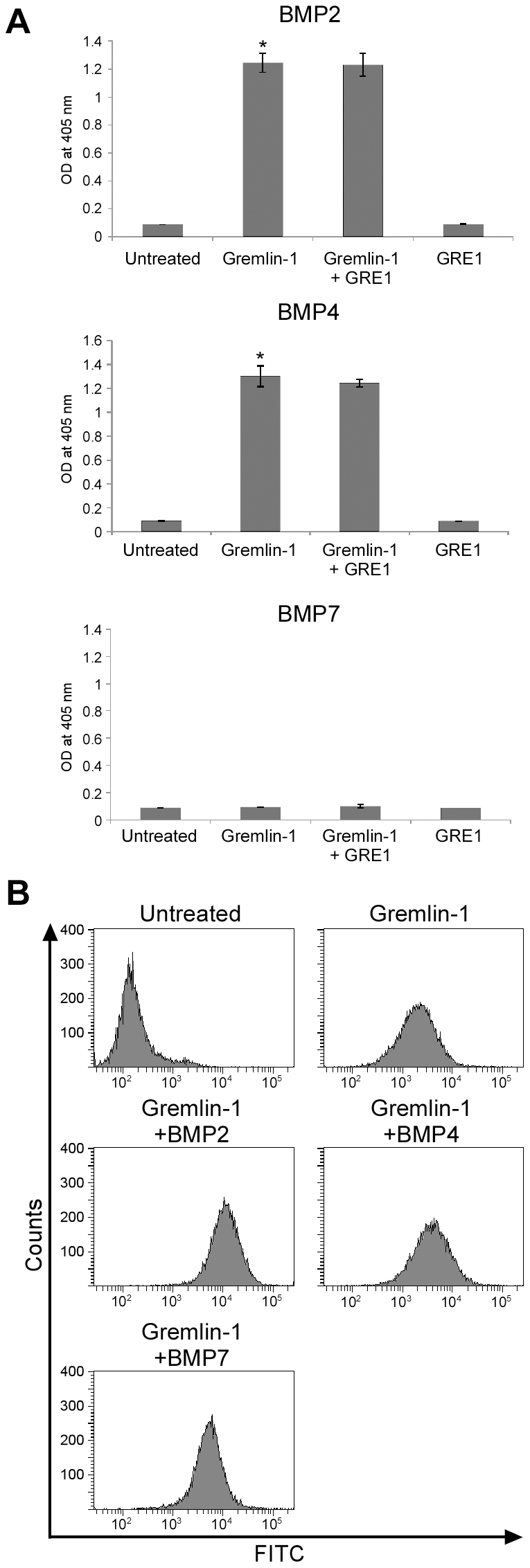
Addition of the neutralizing antibody GRE1 does not interrupt the interaction between gremlin-1 and BMPs. (A) Interaction of gremlin-1 with BMP-2, BMP-4, and BMP-7 as measured by enzyme immunoassay. The neutralizing antibody GRE1 does not affect the interaction between gremlin-1 and BMPs. *P<0.05, Student's *t* test. (B) The interaction of gremlin-1 with A549 cells is unaffected by treatment with a 10 times molar excess of BMP-2, BMP-4, and BMP-7 as measured by flow cytometry.

The most characterized function of gremlin-1 is as a BMP antagonist. Therefore, we investigated the influence of BMPs on the interaction of gremlin-1 with A549 cells. Gremlin-1 forms heterodimers with BMP-2, BMP-4, and BMP-7 and interrupts the binding of BMPs to their receptors. In an enzyme immunoassay, gremlin-1 interacted with BMP-2 and BMP-4. Gremlin-1 did not interact with BMP-7 in our experimental conditions and the reason for this is unclear. Addition of the neutralizing antibody GRE1 did not inhibit the interaction of gremlin-1 with BMP-2 or BMP-4 (Fig. 3A, *P<0.05). In flow cytometric assays, the presence of a molar excess of BMPs did not affect the interaction of gremlin-1 with A549 cells (Fig. 3B). These results indicate that there are likely two separate motifs in gremlin-1 that mediate its interaction with A549 cells and BMPs, and these motifs do not co-localize.

### Gremlin-1 induces A549 cell scattering and migration

**Figure 4:**
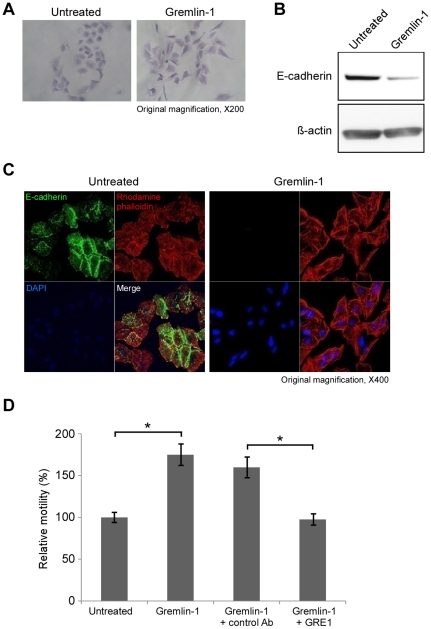
Gremlin-1 induces the scattering and migration of A549 cells *in vitro*. (A) A549 cells appear fibroblast-like after incubation with gremlin-1 for 3 days. (B) E-cadherin protein expression is reduced in A549 cells after incubation with gremlin-1 for 3 days. (C) E-cadherin immunofluorescence (green) in A549 cells is reduced after incubation with gremlin-1 for 3 days. Nuclei were counterstained with DAPI (blue) and actin filaments were counterstained with rhodamine-phalloidin (red). (D) Migration of A549 cells after incubation with gremlin-1 only or gremlin-1 plus GRE1. Addition of the neutralizing antibody GRE1 abolishes gremlin-1 induced migration. **P<0.01, ***P<0.001, Student's *t* test.

When A549 cells were treated with gremlin-1 for 3 days, the cell morphology became fibroblast-like and the cells became scattered (Fig. 4A). E-cadherin expression was markedly reduced in A549 cells cultured with gremlin-1 as evaluated by immunoblot analysis and immunofluorescence staining (Fig. 4B and 4C). In a scratch wound healing assay, treatment with gremlin-1 for 24 h significantly increased the migration of A549 cells (Fig. 4D, ***P<0.001). This effect was completely abolished upon addition of the neutralizing antibody GRE1 (Fig. 4D, **P<0.01).

### Characterization of gremlin-1 transfected A549 cell lines

**Figure 5:**
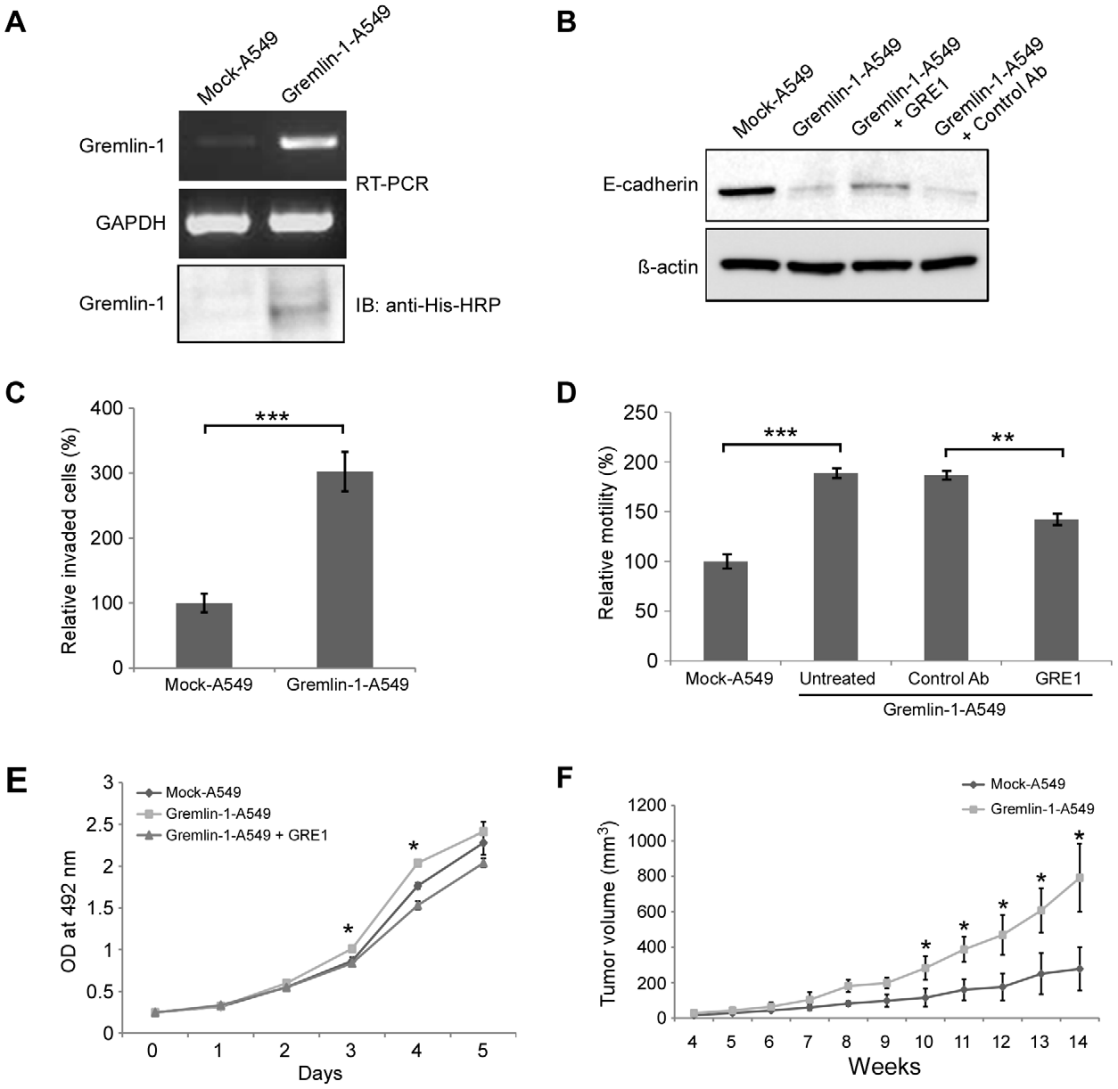
Characterization of gremlin-1-transfected A549 cell lines. (A) RT-PCR and western blot analyses indicate gremlin-1 mRNA and protein are expressed in gremlin-1-A549 cells but not mock-A549 cells. (B) E-cadherin protein expression is reduced in gremlin-1-A549 cells and this effect is attenuated upon addition of the neutralizing antibody GRE1. (C) Gremlin-1-A549 cells show increased invasiveness in cell invasion assays as compared to mock-A549 cells. The cells on the underside of the ECM membrane were stained and counted. ***P<0.001, Student's *t* test. (D) Gremlin-1-A549 cells show increased migration compared to mock-A549 cells and this effect is attenuated upon the addition of the neutralizing antibody GRE1. **P<0.01, ***P<0.001, Student's *t* test. (E) Gremlin-1-A549 cells display an increased growth rate compared to mock-A549 cells as determined by MTS proliferation assay. The neutralizing antibody GRE1 addition blocks this effect. *P<0.05 versus mock-A549, Student's *t* test. (F) Gremlin-1-A549 cells injected subcutaneously in nude mice have an increased rate of tumor growth *in vivo* as compared with injection of mock-A549 cells. Tumor volume is depicted as the average ± standard deviation. *P<0.05 versus mock-A549, Student's *t* test.

Next, we generated stably transfected A549 cell lines containing gremlin-1 (gremlin-1-A549) or empty vector (mock-A549). Using RT-PCR and western blot analyses, we confirmed increased levels of gremlin-1 transcript and protein in the gremlin-1-A549 cells (Fig. 5A). E-cadherin expression was reduced in gremlin-1-A549 cells as compared with mock-A549 cells. However, its expression slightly increased upon addition of the neutralizing antibody GRE1 to the culture media (Fig. 5B). For cell invasion assays, gremlin-1-A549 cells or mock-A549 cells were plated on the upper surface of ECM coated membrane of inner chambers. After 48 h, the cells that migrated through ECM and attached to the bottom of the membrane were stained with crystal violet. We determined that a higher number of gremlin-1-A549 cells migrated as compared to mock-A549 cells (Fig. 5C, ***P<0.001). In a scratch wound healing assay, gremlin-1-A549 cells also showed increased migration as compared with mock-A549 cells, and migration was significantly inhibited upon addition of the neutralizing antibody GRE1 (Fig. 5D, **P<0.01, ***P<0.001). To identify whether gremlin-1 influences cell growth, a cell proliferation assay was performed. Gremlin-1-A549 cells had a higher growth rate compared to mock-A549 cells or untransfected A549 cells (Fig. 5E, *P<0.05). The increased growth rate of gremlin-1-A549 cells was inhibited by the addition of the neutralizing antibody GRE1 (Fig. 5E).

### Gremlin-1 enhances tumor growth in vivo

To evaluate the effect of gremlin-1 on tumorigenesis, gremlin-1-A549 cells or mock-A549 cells were injected subcutaneously into nude mice. Tumor size was measured weekly using a digital caliper. The tumor volume in mice injected with gremlin-1-A549 cells increased more rapidly than those injected with mock-A549 cells, with an approximately 500 mm^3^ difference in tumor volume at 14 weeks post injection (Fig. 5F, *P<0.05). This result suggests that increased expression of gremlin-1 may play a role in tumorigenesis.

## Discussion

Gremlin-1 has a critical role regulating BMPs during embryonic development but its expression is down-regulated in normal adult tissues [7], [8]. Differential display RT-PCR analysis revealed that gremlin-1 is overexpressed in various human tumors including carcinoma of the lung, ovary, kidney, breast, colon, pancreas, and cervix [12]. It was also reported that gremlin-1 is overexpressed in the stroma of basal cell carcinoma (BCC) but not in normal skin according to immunohistochemical analysis. In *in situ* hybridization analyses, elevated gremlin-1 mRNA levels were detected in various cancer tissues, including esophagus, bladder, and prostate [21]. However, the function of gremlin-1 in carcinogenesis has not yet been elucidated.

In this study, we report that gremlin-1 interacts with various cancer cell lines (Fig. 1). Recently it was reported that gremlin-1 interacts with VEGFR2 and induces angiogenic responses *in vitro* and *in vivo* [11]. We evaluated whether VEGFR2 was responsible for the interaction of gremlin-1 with cancer cells. We did not detect VEGFR2 expression in A549 or HeLa cells though both cell lines strongly interacted with gremlin-1 (Fig. 2). Therefore, we conclude that gremlin-1 can bind cancer cells and this binding is not mediated by VEGFR2.

Gremlin-1 is a BMP antagonist that specifically binds to and inhibits the activity of BMP-2, BMP-4, and BMP-7 [4], [5]. BMPs are multi-functional growth factors known to play important roles in morphogenesis and homeostasis of many tissues. In addition, BMP-2, BMP-4, and BMP-7 are frequently overexpressed in various cancers including breast and prostate [22]–[24]. It was reported that BMP-4 reduced the proliferation of BCC cells and addition of gremlin-1 reduced the anti-proliferative effect of BMP-4 indirectly [21]. We verified that the mRNA levels of BMP-2 and BMP-4 were highly expressed in A549 cells (data not shown). We also evaluated the interaction of gremlin-1 with A549 cells in the presence of BMP-2, BMP-4, or BMP-7. We determined that gremlin-1 strongly bound BMP-2 and BMP-4 (Fig. 3A) but this binding did not affect its interaction with cancer cells (Fig. 3B). In addition, the neutralizing antibody GRE1 did not inhibit the binding of gremlin-1 to BMP-2 or BMP-4 (Fig. 3A). Therefore, we conclude the interaction of gremlin-1 with A549 cells is likely mediated by a different motif than the motif involved in the interaction with BMPs.

When A549 cells were incubated with gremlin-1, the cell morphology became fibroblast-like and cells were scattered (Fig. 4A). We also found decreased E-cadherin expression when cells were incubated with gremlin-1 (Fig. 4B, 4C, and 5B). Down-regulation of E-cadherin is associated with epithelial-mescenchymal transition (EMT) and the suppression of E-cadherin [25] and EMT are commonly observed in the progression of cancer [26]. In a scratch wound healing assay, gremlin-1 increased the migration of A549 cells. Addition of the neutralizing antibody GRE1 suppressed the observed increase in migration (Fig. 4D and 5D). Furthermore, gremlin-1-A549 cells showed increased invasion through the ECM-coated membrane. It was previously reported that transfection of A549 cells with gremlin-1 sensitized the cells to EMT upon treatment with TGF-β1 [27]. However, induction of an EMT-like phenotype by gremlin-1 alone has not been reported.

Gremlin-1-A549 cells also showed increased proliferation *in vitro* and *in vivo* (Fig. 5E & 5F). In an *In vitro* setting, the increased proliferation rate was reduced upon addition of the GRE1 antibody. Experiments are currently underway to investigate if gremlin-1 directly influences tumor growth. These data suggest that the secretion of gremlin-1 may increase cell proliferation and thus affect tumorigenesis.

## Supporting Information

Figure S1 **The specificity of the GRE1 antibody.** Western blot analysis (A) and Coomassie staining (B) of the culture supernatant of HEK293F transfected with gremlin-1 indicates that GRE1 reacts specifically with gremlin-1. Lanes 1 and 2 were loaded with culture supernatant of HEK293F cells that were mock-transfected or transfected with gremlin-1, respectively. The gremlin-1 protein has post-translational modification sites and exists in two major forms (glycosylated and unglycosylated) [1].
(TIF)
