# Supplementary figures and images for: Gremlin-1 Induces BMP-Independent Tumor Cell Proliferation, Migration, and Invasion
Source: PLoS One. 2012 Apr 13;7(4):e35100. doi: 10.1371/journal.pone.0035100 (PMC3325980; doi:10.1371/journal.pone.0035100)

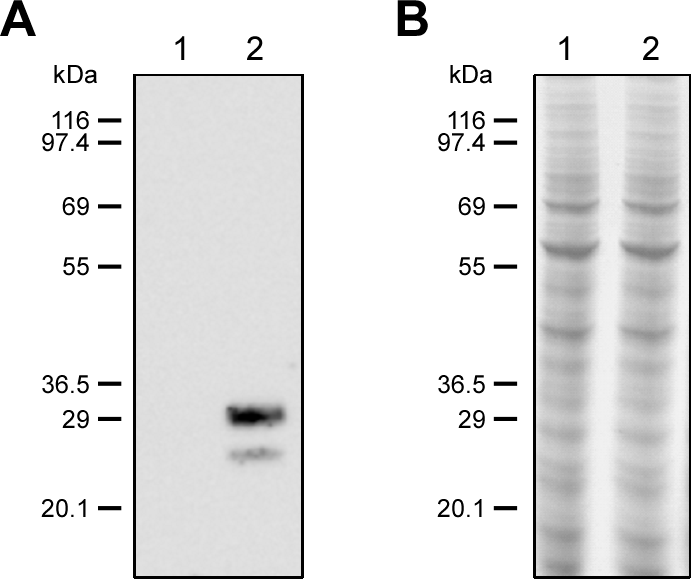

Supplement: Figure S1 — The specificity of the GRE1 antibody. Western blot analysis (A) and Coomassie staining (B) of the culture supernatant of HEK293F transfected with gremlin-1 indicates that GRE1 reacts specifically with gremlin-1. Lanes 1 and 2 were loaded with culture supernatant of HEK293F cells that were mock-transfected or transfected with gremlin-1, respectively. The gremlin-1 protein has post-translational modification sites and exists in two major forms (glycosylated and unglycosylated) [1]. (TIF) [file pone.0035100.s001.tif]
